# *Cancers* Best Paper Award 2015

**DOI:** 10.3390/cancers7020818

**Published:** 2015-05-29

**Authors:** Robert H. Weiss

**Affiliations:** Editor-in-Chief, Division of Nephrology and Cancer Center, University of California, Davis, CA 95616, USA; E-Mail: rhweiss@ucdavis.edu

*Cancers* has instituted a “Best Paper” award to recognize the most outstanding papers in the area of oncology published in *Cancers*. We are pleased to announce the first “*Cancers* Best Paper Award” for 2015. Nominations were selected by the Editor-in-Chief and selected editorial board members from among all the research papers published in 2013 and 2014. We are pleased to announce that the following three papers have won the *Cancers* Best Paper Award in 2015:

First Prize
  **Joëlle Roche, Patrick Nasarre, Robert Gemmill, Aleksander Baldys, Julien Pontis, Christopher Korch, Joëlle Guilhot, Slimane Ait-Si-Ali and Harry Drabkin**
  Global Decrease of Histone H3K27 Acetylation in ZEB1-Induced Epithelial to Mesenchymal Transition in Lung Cancer Cells
  *Cancers* **2013**, *5*(2), 334–356; doi:10.3390/cancers5020334
  Available online: http://www.mdpi.com/2072-6694/5/2/334
  “*Novel findings regarding H3K27 Acetylation during lung cancer EMT*.”
  —Prof. Dr. Afshin Samali
  “*Quite an interesting study on histone acetylation decrease induced by ZEB1 gene. However, it could benefit from more data in patient samples (e.g., expression of target genes)*.”
  —Dr. Jonas Cicenas
Second Prize
  **Mazen A. Juratli, Mustafa Sarimollaoglu, Dmitry A. Nedosekin, Alexander V. Melerzanov, Vladimir P. Zharov and Ekaterina I. Galanzha**
  Dynamic Fluctuation of Circulating Tumor Cells during Cancer Progression
  *Cancers* **2014**, *6*(1), 128–142; doi:10.3390/cancers6010128
  Available online: http://www.mdpi.com/2072-6694/6/1/128
  “*Nice combination of animal model as well as patient data to show fluctuations of circulating tumor cells. It also suggests a potential diagnostic benefit*.”
  —Dr. Jonas Cicenas
  “*Very interesting finding on continuous monitoring of CTCs with significant implications in development of diagnostics*.”
  —Prof. Dr. Afshin Samali
Third Prize
  **Cinzia Mazzini, Pamela Pinzani, Francesca Salvianti, Cristian Scatena, Milena Paglierani, Francesca Ucci, Mario Pazzagli and Daniela Massi**
  Circulating Tumor Cells Detection and Counting in Uveal Melanomas by a Filtration-Based Method
  *Cancers* **2014**, *6*(1), 323–332; doi:10.3390/cancers6010323
  Available online: http://www.mdpi.com/2072-6694/6/1/323
  “Report that the role of CTCs as a negative prognostic marker in uveal melanoma patients after a long-follow up period. These findings have real potential for development.”
  —Prof. Dr. Afshin Samali

**Prize Awarding Committee**
  Editor-in-Chief
  Prof. Dr. Robert H. Weiss
  Division of Nephrology and Cancer Center, University of California, Davis, CA 95616, USA
  E-Mail: rhweiss@ucdavis.edu
Associate Editor
  Dr. Hiromi I. Wettersten
  Department of Medicine, University of California, San Diego, La Jolla, CA 92093, USA
  E-Mail: hirominoue@gmail.com
Editorial Board Member
  Dr. Jonas Cicenas
  CALIPHO Group, Swiss Institute of Bioinformatics, CMU - 1, rue Michel Servet, CH-1211 Geneva 4, Switzerland
  E-Mail: j.cicenas@mapkinases.eu
Editorial Board Member
  Prof. Dr. Sven Perner
  Institute of Pathology, University Hospital of Bonn, Sigmund-Freud-Str. 25, 53127 Bonn, Germany
  E-Mail: sven.perner1972@googlemail.com
Editorial Board Member
  Prof. Dr. Afshin Samali
  Director of Apoptosis Research Centre, School of Natural Sciences, National University of Ireland - Galway, University Road, Galway, Ireland
  E-Mail: afshin.samali@nuigalway.ie

We believe that these three exceptional papers are valuable contributions to *Cancers* and the oncology research field. On behalf of the Prize Awarding Committee and the Editorial Board of *Cancers*, we would like to congratulate these teams for their excellent work. In recognition of their accomplishment, Dr. Joëlle Roche, Dr. Ekaterina I. Galanzha and Dr. Pamela Pinzani will receive the privilege of publishing a paper free of charge in open access format in *Cancers*, respectively, after the usual peer-review procedure.

